# Dispelling Mist That Obscures Positional Vertigo in Vestibular Migraine

**DOI:** 10.3390/brainsci13101487

**Published:** 2023-10-20

**Authors:** E Tian, Fei Li, Dan Liu, Jun Wang, Zhaoqi Guo, Jingyu Chen, Jiaqi Guo, Sulin Zhang

**Affiliations:** 1 Department of Otorhinolaryngology, Union Hospital, Tongji Medical College, Huazhong University of Science and Technology, Wuhan 430022, China; entetian@163.com (E.T.); ld18703212570@163.com (D.L.); ent_wangjun@hust.edu.cn (J.W.); zhaoqiguo1@163.com (Z.G.); chenjingyu0309@163.com (J.C.); jiaqiguo@hust.edu.cn (J.G.); 2 Institute of Otorhinolaryngology, Union Hospital, Tongji Medical College, Huazhong University of Science and Technology, Wuhan 430022, China; 3 Department of Neurology, Changzheng Hospital, Naval Medical University, Shanghai 200003, China; lifei19860711@163.com

**Keywords:** positional nystagmus, vestibular migraine, benign paroxysmal positional vertigo, differential diagnosis, prediction model

## Abstract

(1) Background: Patients with vestibular migraine (VM) often present with positional vertigo. A portion of these patients have features of benign paroxysmal positional vertigo (BPPV). It is a challenge to rapidly identify the BPPV component of VM associated with positional vertigo. (2) Methods: Retrospective data collected from 60 VM and 47 VM + BPPV patients were used to build a diagnostic model, and then prospective data from 47 patients were used for the external validation. All patients had VM manifesting as positional vertigo, with or without accompanying BPPV. The clinical manifestations and the results of vestibular function tests were comprehensively analyzed using logistic regression. (3) Results: The univariate and multivariate analyses showed that the age, symptom duration, tinnitus, ear fullness, nausea, head shaking nystagmus, the direction of the Dix–Hallpike and roll tests, and horizontal gain could help differentiate between the two groups. A nomogram and an online calculator were generated. The C-index was 0.870. The diagnostic model showed good discriminative power and calibration performance during internal and external validation. (4) Conclusions: This study provided a new perspective for diagnosing VM with positional vertigo by identifying the BPPV component and, for the first time, offers a prediction model integrating multiple predictors.

## 1. Introduction

Vestibular migraine (VM) represents a frequent variant of migraine with a recurrent vestibular presentation associated with migraine symptoms (migraine-like headache and/or photophobia and phonophobia and/or visual aura) [1]. It is believed to be one of the most common causes of dizziness. Over the past decades, VM, as a diagnostic entity, has been found to potentially afflict up to 2.7% of the general population [2]. This incidence is higher than the prevalence estimates for benign paroxysmal positional vertigo (BPPV) (1.6%) and Meniere’s disease (0.02–0.5%) [3,4,5].

The vestibular symptoms of VM are characterized by spontaneous vertigo, positional vertigo, visually induced vertigo, and an intolerance to head motion [6,7]. In recent years, research efforts have been directed at the relationship between VM and positional vertigo [8,9,10,11]. When VM presents as positional vertigo, the diagnosis and differentiation pose a significant challenge [8,9]. One reason is that the diagnosis of VM primarily depends on clinical manifestations and currently no diagnostically specific biomarkers are available [12]. Another important factor is that these patients tend to have some other concomitant conditions. Several studies have indicated that the positional vertigo in VM might be attributed to cerebellar dysfunction induced by migraine attacks, or it might stem from aberrant activation of the trigeminal nerve, leading to the release of neuropeptides, such as calcitonin gene-related peptide, which triggers sterile inflammation in the inner ear. These inner ear lesions may result in BPPV secondary to VM [13,14,15,16].

Clinically, positional nystagmus in VM patients cannot be easily traced to a cause of central or peripheral origin. This symptom in such patients tends to persist throughout the entire follow-up period. Their nystagmus is variable and repositioning maneuvers are partially effective. Based on the clinical presentations and possible mechanisms, we speculate that the positional nystagmus observed in these patients may result from a combination of central and peripheral mechanisms (otoconial debris) [17].

The treatment modalities of BPPV and VM differ substantially. If the positional vertigo in a VM patient originates partially from a dislodged otolith, repositioning maneuvers can help relieve BPPV. After removal of the vestibular stimuli that repeatedly trigger VM attacks, anti-migraine treatment can be more effective. However, if the positional vertigo is entirely of central origin, repeated repositioning maneuvers may not achieve therapeutic effect but only exacerbate the patient’s distress, and, in worst cases, even lead to anxiety. Therefore, it is crucial, though challenging, for clinicians to timely identify the BPPV component in VM associated with positional vertigo (VM with PV + BPPV, abbreviated as VM + BPPV).

In this study, we, retrospectively collected the clinical and vestibular function data from VM with PV patients, with focus specifically placed on those with a component of BPPV. We compared these data with those of VM with PV patients without BPPV and established a diagnostic model. In addition, we carried out both internal and external validation. The study aimed to provide a new perspective for the diagnosis of VM with PV by identifying the BPPV component.

## 2. Materials and Methods

### 2.1. Patients and Inclusion/Exclusion Criteria

The model was built by using retrospective data from 107 patients between June 2020 and June 2022, while 47 patients were prospectively included between July 2022 and July 2023 for external validation. All patients presented in the Medical Center for Vertigo and Balance Disorders in Union Hospital affiliated to Tongji Medical College, Wuhan, China. This study proposal was approved by the institutional ethics committee (No. 20210873).

**Figure 1:**
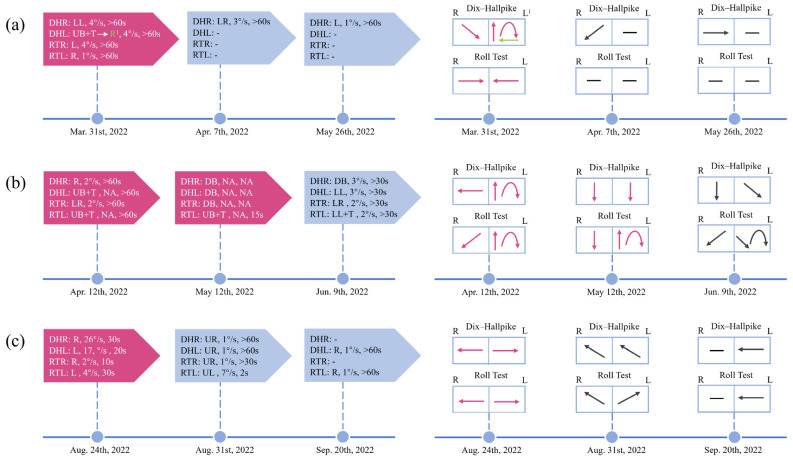
Timeline of the dynamic changes of positional nystagmus in 3 VM + BPPV patients throughout the course of the disease. Changes in positional nystagmus, including direction, SPV, and duration, during the course of the disease (**left**). Visual change in the direction of the patient’s positional nystagmus (**right**). (**a**) Patient 1 underwent one repositioning maneuver. And the typical BPPV nystagmus on the left side disappeared after the treatment. (**b**) Two repositioning maneuvers were performed in patient 2. However, the typical BPPV nystagmus on the left side did not disappear completely and new nystagmus appeared. (**c**) Patient 3 initially presented with typical ageotropic nystagmus. Subsequently, the nystagmus changed and could not be easily localized centrally or peripherally. DHR: Dix–Hallpike right; DHL: Dix–Hallpike left; RTR: roll test right; RTL: roll test left; LL: to the lower left; LR: to the lower right; L: to the left; R: to the right; UB: upbeating; DB: downbeating; T: torsional; UR: to the upper left; UL: to the upper right; -: negative; NA: not available. ^1^ The patient’s nystagmus changed from a mixed vertical (upbeat)-torsional (the upper pole of the eyes beats toward the lower ear) nystagmus for 15 s to persistent rightward nystagmus. The red-colored block indicates that repositioning maneuvers have been performed.

The patients included had clinical manifestations of positional vertigo or nystagmus and all had VM or probable VM according to the diagnostic criteria of the Bárány Society and International Headache Society [12]. They were divided into a VM group and a VM + BPPV group. VM + BPPV patients were diagnosed if separate episodes satisfied the diagnostic criteria for VM and BPPV during the course of the disease (Figure 1c). BPPV was diagnosed if the patient suffered from characteristic nystagmus during positional tests according to the criteria of the Bárány Society [18]. It is important to note that there are five subtypes of BPPV, i.e., posterior canalolithiasis, posterior heavy cupula, lateral canalolithiasis, lateral light cupula, and lateral heavy cupula, and each of these subtypes that met the diagnostic criteria was included [19]. Some patients who were initially thought to have VM did not respond well to prophylactic migraine medication. Among them, positional nystagmus could be partially explained by BPPV. After receiving corresponding semicircular canal repositioning maneuvers, the semicircular canal component of positional nystagmus disappeared and vertigo was relieved in these patients. They were cured by repositioning maneuvers in combination with anti-migraine medication. The above-mentioned patients were also considered to have comorbidities (Figure 1a,b).

The exclusion criteria were (1) severe mobility deficits of the neck; (2) severe visual or auditory impairment; (3) mental disorders; (4) malignancies; (5) severe hepatic or renal dysfunction, metabolic diseases, infectious diseases; (6) age under 18 years; (7) pregnancy; (8) previous vestibular or any other balance disorders; (9) organic disease involving the central nervous system (cerebral infarction or brain tumor, etc.)

### 2.2. Data Gathering and Definitions

All patients were subjected to detailed history taking, otoscopic examination, audiometric tests, and vestibular and neurological examinations. Videonystagmographic (VNG) results with videotape records were collected for spontaneous nystagmus (SN), head shaking nystagmus (HSN), and positional nystagmus. Magnetic resonance imaging (MRI) of the brain and the internal auditory canal was conducted in most patients to rule out the possibility of central lesions. Drug selection was based on indications, age, and possible side effects. During treatment, the patients were followed up at an interval of 1 week. After the completion of their treatment, patients were followed up once every month for 3 months, and then once every 3 months.

The sample size was determined by the available data during the study period. Baseline clinical and vestibular function examination data were harvested from outpatients (the specific operation methods of positional tests, VNG, and vestibular autorotation test are provided in the Supplementary Material) [20,21,22,23]. If positional nystagmus was horizontal or vertical (upbeat)-torsional with the upper pole of the eyes beating toward the lower ear [18], the nystagmus might be taken as being of a semicircular canal (SC) origin. The other kinds of positional nystagmus were from other sources rather than SC. Each predictor was measured independently.

### 2.3. Statistical Analysis

Based on the available data and our clinical experience, a pre-defined set of potentially relevant predictors of the outcome were selected. The data were collected, stored using EpiData 3.1 and analyzed by R 4.2.2 and IBM SPSS 26.0. Continuous data were presented as means ± standard deviations, and categorical data were expressed as percentages. The Shapiro–Wilk test was conducted to assess the normality of the data. The Student t-test was employed to evaluate continuous variables. We compared the differences in the rates of clinical symptoms and abnormal vestibular function test results between the two groups using the chi-square test or Fisher’s exact test. Some data were missing (Supplementary Material, Figure S1). We made a multiple imputation model in the R (4.2.2) software. The SPV and duration of the positional test were removed based on the variance inflation factor to avoid the multicollinearity. Univariate and multivariate analyses were performed to screen for diagnostic variables. Internal and external bootstrap validation was performed. The calibration of the nomogram was evaluated by the Hosmer–Lemeshow test for the goodness of fit (GOF) [24]. The discrimination and predictive power were assessed in terms of the concordance index (C-index), receiver operating characteristic (ROC) curve, and area under curve (AUC).

## 3. Results

### 3.1. Demographic and Clinical Characteristics

The characteristics of the 107 patients in the development cohort and the 47 patients in the validation cohort are shown in Table S1. No difference was found in the original dataset and the imputed dataset (Figure S2), and the baseline clinical and ancillary examination data of the VM and VM + BPPV groups in the development cohort are listed in Table 1. The majority of the patients were women in both groups. The mean age was 49.4 ± 12.8 years in the VM group, and 53.5 ± 10.0 years in the VM + BPPV group. The duration of positional vertigo (*p* = 0.038) was shorter in the VM + BPPV group. The incidence of nausea was lower (*p* = 0.021) and the rate of HSN (*p* = 0.030) was higher in the VM + BPPV group compared to the VM group. The positional test revealed a significant distinction in the nystagmus characteristics between the two groups. Although the three parameters of the vestibular autorotation test (VAT) were not significantly different between the VM and VM + BPPV groups, the abnormal horizontal gain was more suggestive of VM.

### 3.2. Model Establishment and Visualization

**Figure 2:**
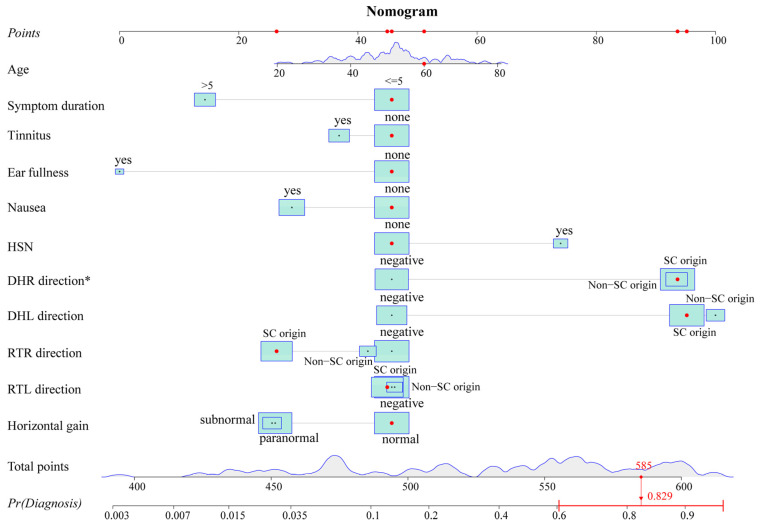
Nomogram. The nomogram predicts the diagnostic probability for the 100th patient of the dataset. Red dots represent the value of this predictor for this patient. The total score can be obtained by adding up the scores corresponding to these red dots. The predicted probability can be calculated from the total score. The probability of this patient is 0.829 from the graph and is greater than 0.471, indicating that the patient is more likely to have VM + BPPV. HSN: head shaking nystagmus; DHR: Dix–Hallpike right; DHL: Dix–Hallpike left; RTR: roll test right; RTL: roll test left; SC: semicircular canal; Pr: probability. * *p* < 0.05.

The variables with *p*-values of <0.2 in the univariate analysis were fitted into the multivariate logistic regression model. The unadjusted associations of the univariate and adjusted associations of the multivariate analysis results are given in Table 2. The regression coefficient and odds ratio (OR) were also reported. A nomogram (Figure 2) was created by using the variables in the multivariable model, including age, symptom duration, tinnitus, ear fullness, nausea, HSN, Dix–Hallpike right (DHR) direction, Dix–Hallpike left (DHL) direction, roll test right (RTR) direction, roll test left (RTL) direction, and horizontal gain. To obtain an easy-to-use tool, an online calculator (https://tiane.shinyapps.io/dynamic_nomogram/, accessed on 14 October 2023, Figure S3) was also created. Using the online calculator, one can simply enter the clinical variables to predict a patient’s diagnostic probability. 

### 3.3. Model Validation

**Figure 3:**
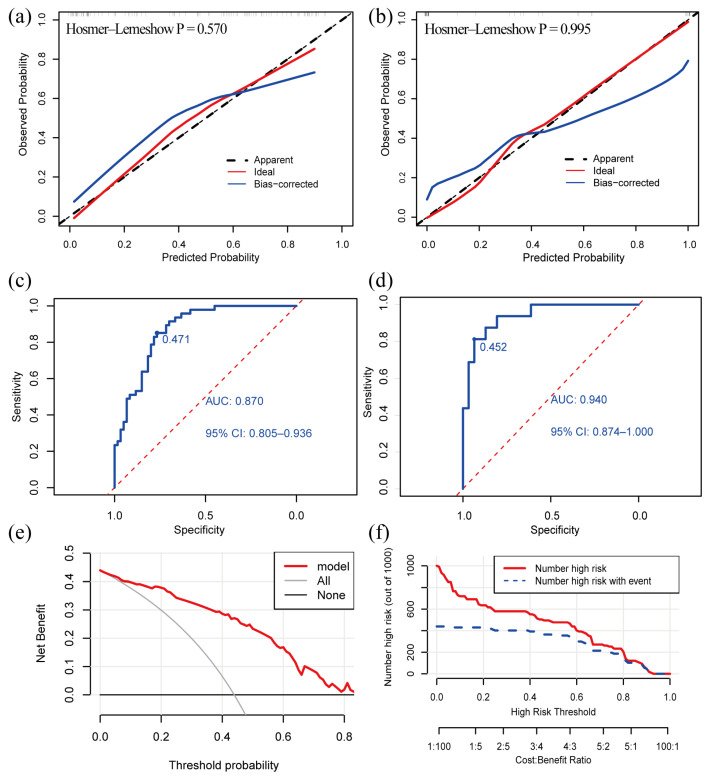
Validation and clinical utility of the diagnostic model. (**a**) Calibration curve of development cohort; (**b**) calibration curve of validation cohort; (**c**) ROC curve of development cohort; (**d**) ROC curve in validation cohort; (**e**) DCA reveals that the nomogram provides a net benefit better than that of treating everyone (the grey line) or treating no one (the black line), with a broad range of probability threshold; (**f**) CIC. The red line indicates the number of people who were judged by the model to be at high risk for different probability thresholds. The blue line denotes the number of people who were judged by the model to be at high risk and actually had an outcome under a given probability threshold. A cost: benefit ratio is also at the bottom, denoting the cost-to-benefit ratio under different probability thresholds. ROC: receiver operating characteristic; AUC: the area under curve; DCA: decision curve analysis; CIC: clinical impact curve.

The C-index of the model was 0.870 (95% confidence interval: 0.805–0.935) in the development cohort and 0.940 (95% confidence interval: 0.876–1.000) in the validation cohort, indicating that the model had a good discriminative power. The calibration curve exhibited that the predictive curves were close to the ideal curve and the *p*-value of the GOF test was 0.570 and 0.995, which suggests that the calibration was acceptable and the predicted value of the model was comparable to the true value (Figure 3a,b).

The Youden index obtained from the ROC analyses was used to determine the optimal cutoff value. The AUC value of our model was 0.870 and the cutoff value was 0.471 in the development cohort (Figure 3c). The AUC value of our model was 0.940 in the validation cohort (Figure 3d). The ROC curves demonstrated a good discriminative power of the models. A patient is more likely to suffer from VM + BPPV if the predicted probability obtained from the nomogram or online calculator is higher than 0.471, and, otherwise, the patient is more likely to have VM.

### 3.4. Clinical Utility

We created a decision curve analysis (DCA) and a clinical impact curve (CIC) to assess the clinical utility of our model. The DCA curve showed that the net benefit was higher than the two extreme curves over a broad range of threshold probabilities, which demonstrated the model had a good clinical utility (Figure 3e). The CIC predicted the probability stratification of 1000 subjects (Figure 3f) and demonstrated that the nomogram possessed a strong predictive power since the model had an excellent overall net benefit within a wide and practical range of threshold probabilities.

## 4. Discussion

In this study, we successfully achieved the goal of identifying the BPPV component in the VM patients with PV by constructing a nomogram. And the nomogram was validated both internally and externally. Our analyses found that the age, symptom duration, tinnitus, ear fullness, nausea, HSN, DHR direction, DHL direction, RTR direction, RTL direction, and horizontal gain were determinants that distinguish between VM and VM + BPPV. The ROC curve indicated that the model had a high accuracy in correctly distinguishing between the two groups and the calibration curve showed that the predicted values of the model were close to the actual values. Moreover, the DCA curve and CIC suggested good clinical practicability of the nomogram.

Patients in both groups had a history of migraine or concomitantly suffered from various migraine symptoms, such as blurred vision, fear of bright lights or feeling irritated by noise during vertigo attacks. HSN, which is typically associated with peripheral vestibulopathy but can also be concomitant with central disorders, is caused by the central velocity storage mechanism that amplifies peripheral vestibular asymmetry [25]. It was possibly, for this reason, that HSN was more common in VM + BPPV patients. The positional nystagmus in the two groups was mostly of SC origin but not typical BPPV nystagmus. If a patient’s nystagmus is induced by the Dix–Hallpike test, the patient may have VM + BPPV since BPPV is more commonly found in the posterior canal. In the roll test, the nystagmus of SC origin is more likely to be VM. Furthermore, the patients with VM + BPPV were more likely to have compound nystagmus and a number of those patients experienced frequent nystagmus direction changes (Figure 1). Additionally, our research provided evidence for the possible clinical application of VAT. The horizontal gain can be used as an indicator that helps differentiate between VM and VM + BPPV.

For VM + BPPV, the pathogenesis is still not clear, and we hereby propose the following possible mechanisms.

### 4.1. VM Mimics BPPV

Vestibular migraine is a chameleon of dizziness and can mimic a variety of vestibular disorders, including BPPV [26]. In this scenario, VM presents a genuine threat, and the repositioning maneuver is ineffective. 

According to King’s research, the vestibular symptoms of VM start from the vestibular nuclei [27]. The vestibular nuclei are a node in a negative feedback loop. They project to (and could sensitize) the cerebellar nodules and uvula, which then suppress vestibular nuclei activity through reciprocal inhibitory projections. The brain’s ability to generate accurate estimates of motion and orientation is aided by this feedback loop. In patients with VM, a migraine episode may sensitize the vestibular nuclei. In such circumstances, the inhibition of the nodules and uvula is no longer sufficient to maintain stability, especially when the activity of the nodules and uvula is modulated by altered head orientation, resulting in episodes of vestibular dysfunction [14,27,28]. Those attacks are associated with migraine and are triggered by head tilting (e.g., “positional”) [9]. In some situations, the positional vertigo and nystagmus may resemble BPPV.

### 4.2. VM and BPPV Episodes

A family history of VM suggests that the onset of VM may have a genetic origin. It was discovered that some families with hemiplegic migraine had mutations in the gene encoding the α1A transmembrane subunit of a voltage-gated calcium channel [29]. This gene belongs to a sizable gene family that produces a number of subunits of voltage-gated neuronal calcium channels, some of which are expressed in neurons and hair cells. Ion channels play a crucial role in the inner ear’s ability to maintain resting potentials and excite primary afferent neurons. The brain and inner ear symptoms in patients with migraine and BPPV may be ascribed to mutations in ion channel genes that are shared by the brain and inner ear [17]. 

### 4.3. VM Causes BPPV Attack

Given that vasospasm is a well-recognized migraine symptom, vasospasm of the labyrinthine arteries is potentially a mechanism underlying migraine. Etiologically, the release of otoconia from the macular membrane is thought to be the cause of BPPV, and, in fact, is a well-researched aftereffect of ischemic damage to the inner ear [30]. It is reasonable to speculate that migraine patients are more likely to experience recurrent episodes of BPPV because they are repeatedly subjected to damage to their inner ears (possibly as a result of vasospasm or other mechanisms) [17]. Another mechanism proposed to account for the pathophysiological relationship between migraine and vertigo is trigeminovascular activation. Through reciprocal connections with the vestibular nuclei, trigeminal activation may cause vestibular symptoms during a migraine attack [15,31]. In addition, trigeminal nerve stimulation has also been found to cause fluid extravasation in the cochlea [32]. It may, theoretically, cause the otoconia to detach from the otolith organs.

### 4.4. BPPV Causes VM Attack

Another theory proposes that the vasospastic mechanism of migraine is a secondary phenomenon due to some underlying metabolic disorders [17]. For instance, vasospasm in the inner ear might take place after a primary metabolic abnormality in the inner ear. An electronystagmographic study showed that in patients presenting with vertigo, peripheral vestibular abnormalities were more frequent in migraine patients than in non-migraineurs [33]. In Murdin L’s research, the vertigo elicited by vestibular stimulation could act as a migraine trigger [34]. Another study showed that the prevalence of migraine was higher than expected in patients with BPPV [3]. Murdin L’s findings further pointed to a potential contributing mechanism: BPPV episodes may exert an unmasking effect on migraine, serving as triggers in those who are vulnerable, thereby increasing the frequency of migraine attacks [34].

Another specific condition, residual dizziness, is also worth mentioning. After successful repositioning maneuvers, some BPPV patients may experience lightheadedness, transient unsteadiness or persistent non-positional vertigo of various duration [35]. This is more common among elderly patients and those with anxiety disorders [36,37,38]. The complexity and protracted course of the disease in these patients may make it tough to distinguish between VM and VM + BPPV, and careful differentiation is required.

**Figure 4:**
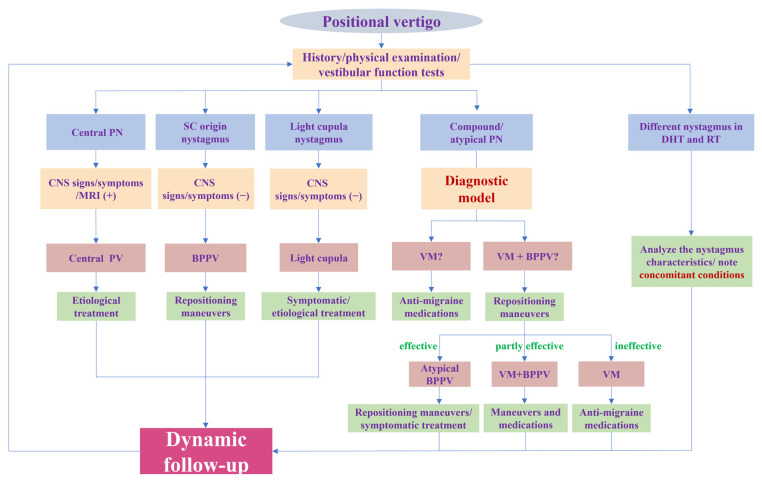
Flowchart of diagnosis and treatment for positional vertigo. PN: positional nystagmus; SC: semicircular canal; DHT: Dix–Hallpike test; RT: roll test; CNS: central nervous system; PV: positional vertigo; BPPV: benign paroxysmal positional vertigo; VM: vestibular migraine.

To sum up, our research provides a new perspective for the diagnosis of VM with PV by identifying the BPPV component. Furthermore, we offer a rapid and convenient tool for identifying the BPPV component by establishing a diagnostic model. All variables included in the model are readily available clinically. A physician can obtain a predicted probability by simply imputing the variables into the online calculator (https://tiane.shinyapps.io/dynamic_nomogram/, accessed on 14 October 2023). If the predicted probability is higher than 0.471, the patient is more likely to have VM + BPPV, and, otherwise, the patient is more likely to have VM. To the best of our knowledge, this is the first prediction model to diagnose these two groups by integrating both the clinical features and results of vestibular tests. For VM with PV patients, it is important to correctly identify the BPPV component and avoid repeated repositioning maneuvers. However, for patients with VM + BPPV, a timely repositioning maneuver is more conducive to their recovery, as it eliminates the peripheral vestibular stimulation that triggers VM attacks. More importantly, we should pay attention to the patient’s responses to treatment and conduct follow-ups to dynamically confirm or modify the diagnosis (Figure 4).

This study is subject to several limitations. First, our study used a single-center retrospective cohort of small size and prospective studies with larger samples are warranted. Second, the results of the vestibular function tests used in this study may not be readily available to community physicians, which may limit the clinical applicability of this model. However, our research is aimed at diagnosing patients with difficult to differentiate positional vertigo. Most of these patients will eventually seek care in tertiary hospitals where the vestibular function tests mentioned in our model can be performed. Therefore, our research still has significant clinical applicability.

We plan to broaden and deepen our study in the future to further enhance its clinical relevance and applicability. Firstly, we will collect more external validation data to further confirm the applicability of our model in different populations, thus improving its clinical applicability. Secondly, we intend to explore personalized interventions based on the model predictions to optimize the treatment of patients with VM and VM + BPPV. Finally, we will endeavor to work out strategies to seamlessly integrate our diagnostic model into clinical practice.

## 5. Conclusions

We have, for the first time, established and validated a diagnostic model that can distinguish between VM and VM + BPPV. The model performs well in terms of calibration and discrimination. Tests for the age, symptom duration, tinnitus, ear fullness, nausea, HSN, Dix–Hallpike and roll test directions, and horizontal gain are incorporated into the model to differentiate between VM and VM + BPPV. All these indicators are easy to measure clinically. The nomogram and online calculator provide easy-to-use tools for clinicians. We hope that this fast and convenient tool will help to accurately identify the BPPV component in VM with PV, allowing for the better selection of appropriate and effective treatment modalities.

## Figures and Tables

**Table 1:** The baseline demographics and clinical features of VM and VM + BPPV patients.

	VM *n* = 60 (56.1%)	VM + BPPV *n* = 47 (43.9%)	*p*-Value
Gender (female) (%)	44 (73.3)	38 (80.9)	0.362
Age (range), year	49.4 ± 12.8	53.5 ± 10.0	0.070
Symptom duration (min)			0.038
≤5	39 (65.0)	39 (83.0)	
>5	21 (35.0)	8 (17.0)	
Cochlear symptoms (%) ^1^			
Hearing loss ^2^	12 (20.0)	11 (23.4)	0.671
Tinnitus	19 (31.7)	9 (19.1)	0.144
Fullness	5 (8.3)	1 (2.1)	0.336
Nausea (%)	27 (45.0)	11 (23.4)	0.021
Migrainous symptoms (%) ^1^			
Headache	28 (46.7)	17 (36.2)	0.275
Phonophobia	12 (20.0)	6 (12.8)	0.321
Photophobia	10 (16.7)	5 (10.6)	0.373
Visual aura	3 (5.0)	1 (2.1)	0.792
Family history (%)	9 (15.0)	4 (8.5)	0.308
SN (%)	15 (25.0)	10 (21.3)	0.651
HSN (%)	5 (8.3)	11 (23.4)	0.030
DHR direction (%)			<0.001
Negative	38 (63.3)	5 (10.6)	
SC origin	16 (26.7)	30 (63.8)	
Non-SC origin	6 (10.0)	12 (25.6)	
DHR SPV (°/s)			<0.001
0	38 (63.3)	5 (10.6)	
0–5	16 (26.7)	27 (57.5)	
≥5	6 (10.0)	15 (31.9)	
DHR duration (s)			<0.001
0	38 (63.3)	5 (10.6)	
0–60	4 (6.7)	23 (48.9)	
≥60	18 (30.0)	19 (40.5)	
DHL direction (%)			<0.001
Negative	36 (60.0)	4 (8.5)	
SC origin	19 (31.7)	33 (70.2)	
Non-SC origin	5 (8.3)	10 (21.3)	
DHL SPV (°/s)			<0.001
0	36 (60.0)	4 (8.5)	
0–5	13 (21.7)	24 (51.1)	
≥5	11 (18.3)	19 (40.4)	
DHL duration (°/s)			<0.001
0	36 (60.0)	4 (8.5)	
0–60	7 (11.7)	19 (40.4)	
≥60	17 (28.3)	24 (51.1)	
RTR direction (%)			<0.001
Negative	40 (66.7)	12 (25.5)	
SC origin	16 (26.7)	27 (57.4)	
Non-SC origin	4 (6.6)	8 (17.1)	
RTR SPV (°/s)			<0.001
0	40 (66.7)	12 (25.5)	
0–5	13 (21.7)	27 (57.4)	
≥5	7 (11.6)	8 (17.1)	
RTR duration (°/s)			<0.001
0	40 (66.7)	12 (25.5)	
0–60	5 (8.3)	13 (27.7)	
≥60	15 (25.0)	22 (46.8)	
RTL direction (%)			<0.001
Negative	40 (66.7)	12 (25.5)	
SC origin	17 (28.3)	27 (57.5)	
Non-SC origin	3 (5.0)	8 (17.0)	
RTL SPV (°/s)			<0.001
0	40 (66.7)	12 (25.5)	
0–5	12 (20.0)	28 (59.6)	
≥5	8 (13.3)	7 (14.9)	
RTL duration (°/s)			<0.001
0	40 (66.7)	12 (25.5)	
0–60	6 (10.0)	17 (36.2)	
≥60	14 (23.3)	18 (38.3)	
Horizontal gain			0.130
Normal	22 (36.7)	26 (55.3)	
Paranormal	28 (46.7)	17 (36.2)	
Subnormal	10 (16.6)	4 (8.5)	
Horizontal phase			0.776
Normal	22 (36.7)	20 (42.6)	
Paranormal	36 (60.0)	25 (53.2)	
Subnormal	2 (3.3)	2 (4.2)	
Asymmetry	12 (20.0)	8 (17.0)	0.695

^1^ The following symptoms might co-exist. ^2^ Hearing loss was symmetrical and mild to moderate in both ears with a past medical history.

**Table 2:** Univariate and multivariate logistic analyses for VM and VM + BPPV patients.

	Univariate Analysis			Multivariate Analysis		
	β	OR (95% CI)	*p*	β	OR (95% CI)	*p*
Gender (female) (%)	−0.429	0.651 (0.250–1.617)	0.364	-	-	-
Age (range), year	0.031	1.032 (0.998–1.069)	0.073	0.024	1.023 (0.977–1.074)	0.323
Symptom duration (%)	−9.651	0.381 (0.144–0.935)	0.041	−1.199	0.302 (0.070–1.182)	0.092
Cochlear symptoms (%) ^1^						
Hearing loss ^2^	0.200	1.222 (0.479–3.099)	0.670	-	-	-
Tinnitus	−0.671	0.511 (0.199–1.240)	0.147	−0.338	0.713 (0.189–2.634)	0.610
Fullness	−1.431	0.239 (0.012–1.552)	0.199	−1.747	0.174 (0.004–2.850)	0.275
Nausea (%)	−0.985	0.373 (0.156–0.853)	0.022	−0.641	0.527 (0.147–1.817)	0.312
Migrainous symptoms (%) ^1^						
Headache	−0.434	0.648 (0.293–1.408)	0.276	-	-	-
Phonophobia	−0.536	0.585 (0.189–1.649)	0.324	-	-	-
Photophobia	−0.519	0.595 (0.174–1.814)	0.376	-	-	-
Visual aura	−0.884	0.413 (0.020–3.347)	0.450	-	-	-
Family history (%)	−0.640	0.527 (0.135–1.741)	0.314	-	-	-
SN (%)	−0.210	0.811 (0.318–1.999)	0.652	-	-	-
HSN (%)	1.212	3.361 (1.123–11.420)	0.037	1.084	2.956 (0.687–14.716)	0.160
DHR direction (%)						
Negative	Reference			Reference		
SC origin	2.657	14.250 (5.024–48.023)	<0.001	1.834	6.260 (1.283–35.109)	0.027
Non−SC origin	2.721	15.200 (4.198–64.810)	<0.001	1.828	6.223 (0.956–44.061)	0.057
DHL direction (%)						
Negative	Reference			Reference		
SC origin	2.749	15.6312 (5.283–58.448)	<0.001	1.894	6.643 (0.874–60.445)	0.075
Non−SC origin	2.890	18.000 (4.402–90.461)	<0.001	2.078	7.986 (0.840–86.465)	0.072
RTR direction (%)						
Negative	Reference			Reference		
SC origin	1.727	5.625 (2.356–14.190)	<0.001	−0.739	0.478 (0.072–2.701)	0.417
Non−SC origin	1.897	6.667 (1.787–28.803)	0.006	−0.154	0.858 (0.067–10.653)	0.904
RTL direction (%)						
Negative	Reference			Reference		
SC origin	1.667	5.294 (2.233–13.238)	<0.001	−0.029	0.972 (0.174–4.707)	0.972
Non−SC origin	2.185	8.889 (2.201–45.755)	0.003	0.018	1.019 (0.097–11.856)	0.988
Horizontal gain						
Normal	Reference			Reference		
Paranormal	−0.666	0.514 (0.222–1.168)	0.115	−0.748	0.473 (0.145–1.452)	0.198
Subnormal	−1.083	0.338 (0.083–1.166)	0.100	−0.768	0.464 (0.057–3.147)	0.444
Horizontal phase						
Normal	Reference			Reference		
Paranormal	−0.269	0.764 (0.345–1.689)	0.505	-	-	-
Subnormal	0.095	1.100 (0.123–9.877)	0.927	-	-	-
Asymmetry	−0.198	0.821 (0.295–2.184)	0.695	-	-	-

^1^ The following symptoms might co-exist. ^2^ Hearing loss was symmetrical and mild to moderate in both ears with a past medical history. β: regression coefficient; OR: odds ratio; CI: confidence interval.

## Data Availability

The data presented in this study are available on request from the corresponding author. The data are not publicly available for privacy and ethical reasons.

## Supplementary Materials

The following supporting information can be downloaded at: https://www.mdpi.com/article/10.3390/brainsci13101487/s1, Figure S1: The missingness map of the data in development cohort and validation cohort; Figure S2: Comparison of datasets before and after multiple imputation in development cohort; Figure S3: The online calculator of nomogram; Table S1: Comparison of datasets in development cohort and validation cohort.
