# Supplementary material for: Dispelling Mist That Obscures Positional Vertigo in Vestibular Migraine
Source: Brain Sci. 2023 Oct 20;13(10):1487. doi: 10.3390/brainsci13101487 (PMC10605638; doi:10.3390/brainsci13101487)
Supplement: Supplementary file 1 [file brainsci-13-01487-s001.zip › brainsci-2658481-supplementary.pdf]

Dispelling Mist That Obscures Positional Vertigo in Vestibular Migraine

E Tian [1,2,†], Fei Li [3,†], Dan Liu [1,2], Jun Wang [1,2], Zhaoqi Guo [1,2], Jingyu Chen [1,2], Jiaqi Guo [1,2] and Sulin Zhang [1,2,*]

1 Department of Otorhinolaryngology, Union Hospital, Tongji Medical College, Huazhong University of Science and Technology, Wuhan 430022, China
2 Institute of Otorhinolaryngology, Union Hospital, Tongji Medical College, Huazhong University of Science and Technology, Wuhan 430022, China
3 Department of Neurology, Changzheng Hospital, Naval Medical University, Shanghai 200003, China
* Correspondence: sulin_zhang@hust.edu.cn
† These authors contributed equally to this work.

Methods

1. Positional tests and Videonystagmographic (VNG)

Dix-Hallpike and Roll test were performed as described previously [1,2]. During the tests, the examiner asked the patient if he or she had vertigo and nystagmus. If nystagmus was present, the intensity and direction were recorded. The patient was observed for at least 1 minute or until the nystagmus was gone.

VisualEyes™ VNG (Micromedical Technologies Inc., Chatham, IL, USA) is a vestibular assessment technique. It allows an examiner to observe and record eye movements in real time. In our study, patients wore infrared video nystagmus eye shields to check for SN, HSN and positional nystagmus. Infrared photography and digital video imaging were used to isolate the location of the pupil so that trajectories of eye movements could be generated [3]. The main observation indicators included the direction, intensity and duration of nystagmus. The nystagmus intensity was measured in terms of the slow phase velocity (SPV).

2. Vestibular autorotation test (VAT)

The VAT developed by WSR (Western System Research, Pasadena, CA, USA) was used for the examination. Eye movement data were recorded with an electronystagmographic (ENG) amplifier and an angular velocity sensor attached on the head [4]. The subjects took a sitting position. Before test, the skin where the electrodes were to be placed was cleaned with alcohol. Two electrodes were placed in the outer canthus of each eye. Another pair of electrodes were positioned above and below the left eye, and the last one was put above the middle of both eyebrows. During the test, patients were asked to fix their eyes on a target 120 cm away and perform head movements on the horizontal and vertical planes as

instructed. Gain, phase and asymmetry values for eye movements in the horizontal plane, and gain, phase for eye movements on the vertical plane were obtained. The gain is, by definition, the ratio of head velocity to eye velocity. The position of the eyes in relation to the head is referred to as phase. The eyes' deviation from the middle line during a horizontal head rotation is taken as asymmetrical. A software package (VATPLUS®) was utilized to record, analyze, screen and store data for each detection. Final data collection covered three parameters: horizontal gain, horizontal phase and asymmetry.

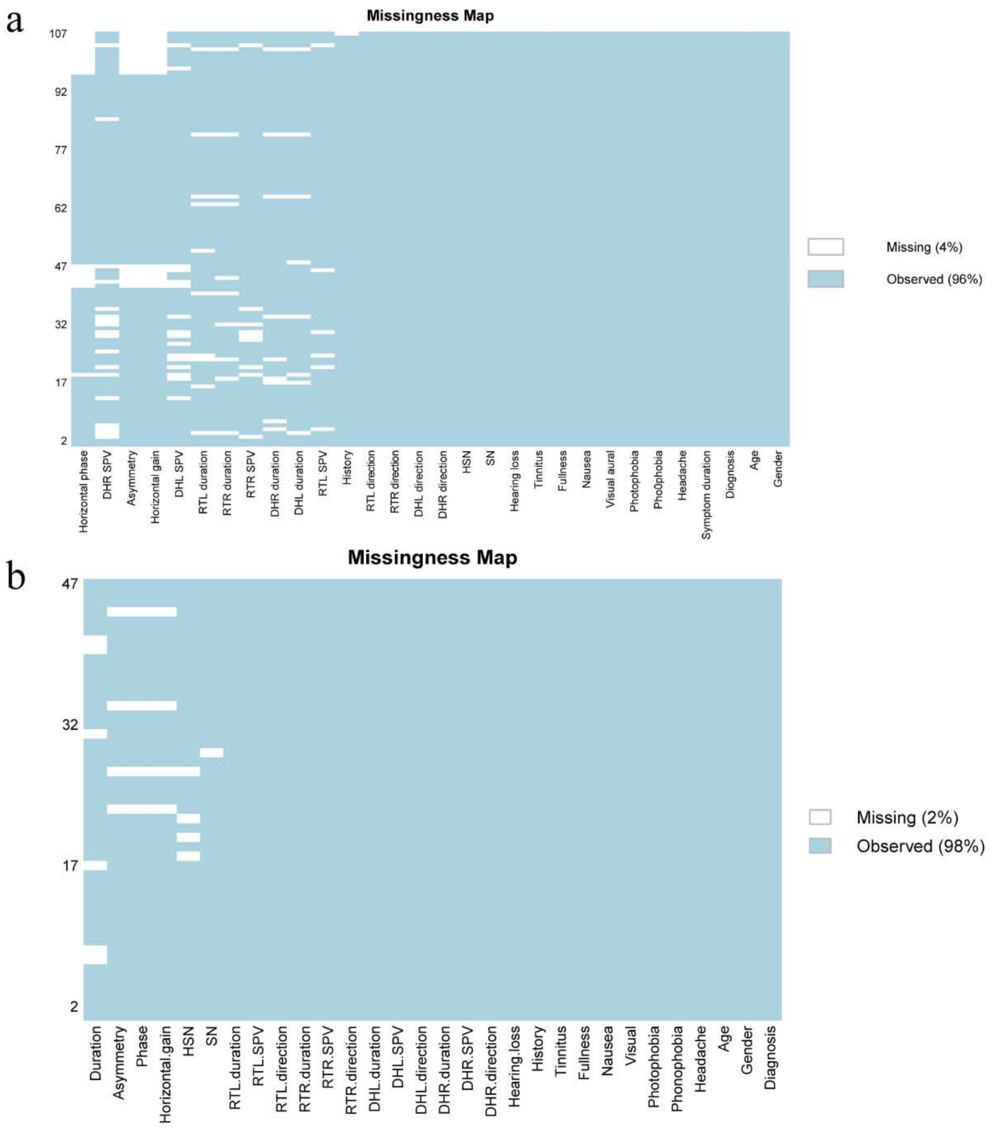

Figure S1. The missingness map of the data in development cohort (a) and validation cohort (b).

Missing values are shown in white. Our data is missing no more than 20% of each item.

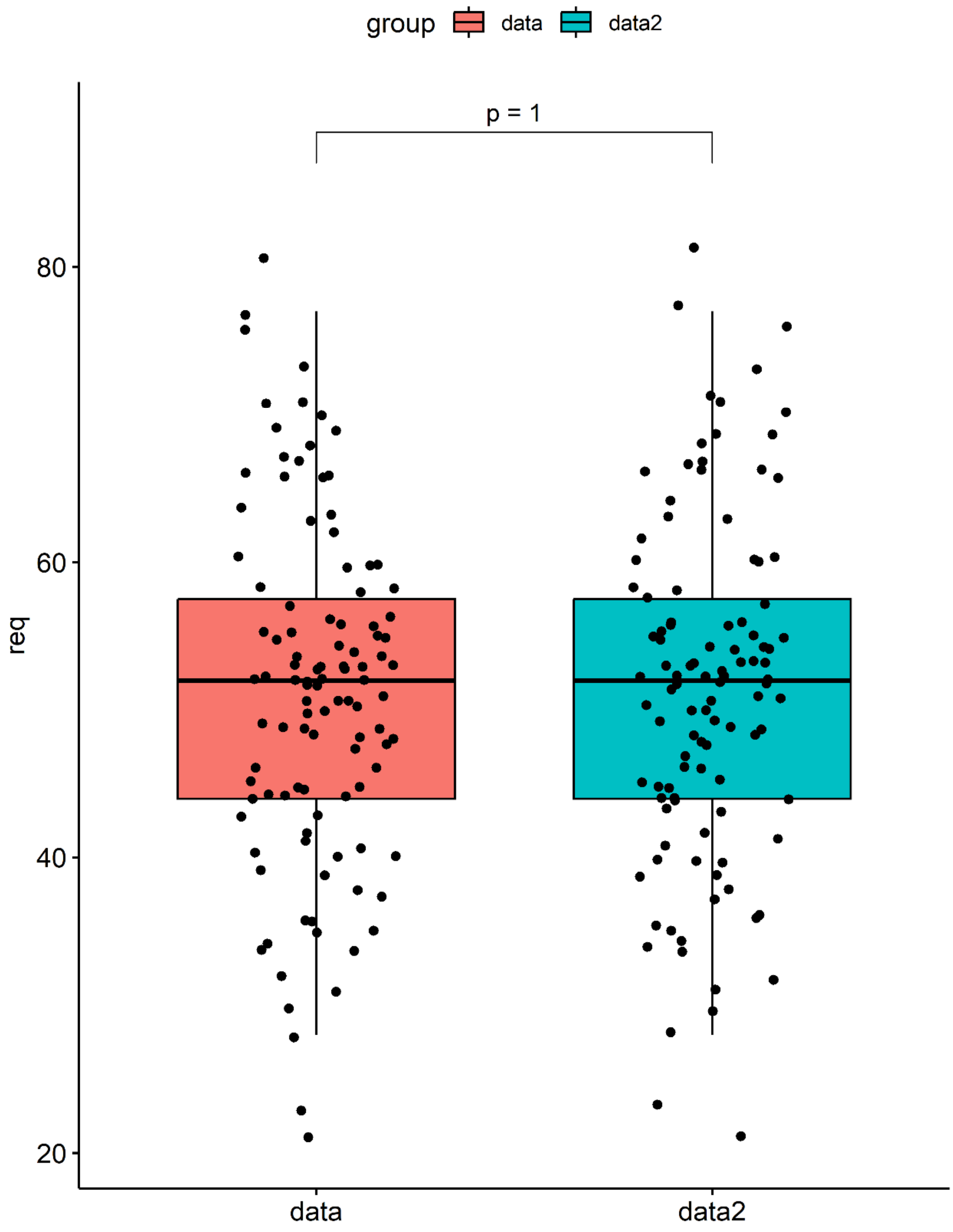

Figure S2. Comparison of datasets before and after multiple imputation in development cohort.

data: the original dataset. data2: the imputed dataset.

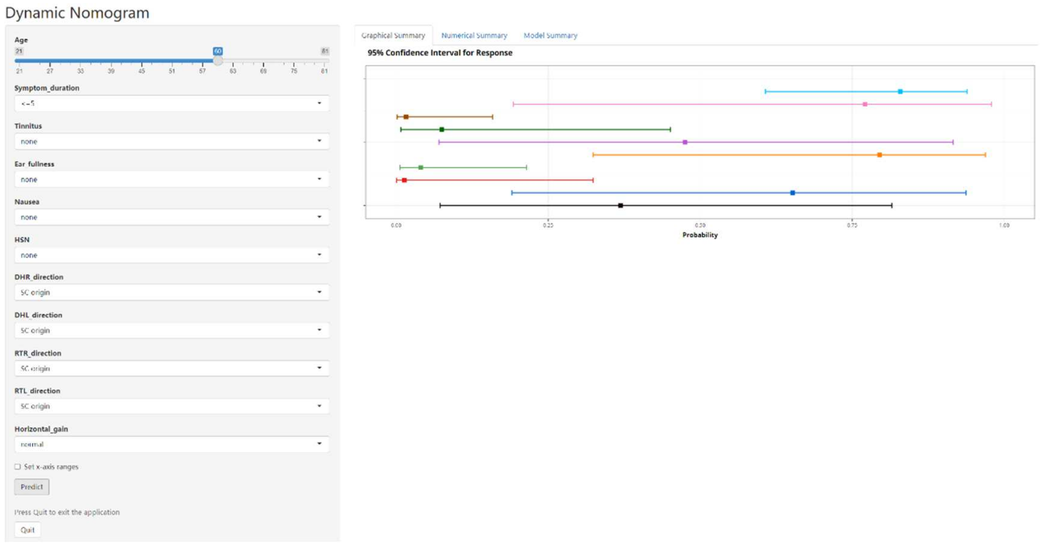

Figure S3. The online calculator of nomogram.

(https://tiane.shinyapps.io/dynamic_nomogram/**)**

Table S1. Comparison of datasets in development cohort and validation cohort.

	Development cohort n = 147	Validation cohort n = 47	*P*
Gender (female) (%)	76.6	66.0	0.167
Age (range), year	51.19 ± 11.76	48.96 ± 11.73	0.676
Symptom duration (min)			0.092
≤ 5	72.9	80.9	
> 5	27.1	19.1	
Cochlear symptoms (%)[1]			
Hearing loss[2]	21.5	10.6	0.108
Tinnitus	26.2	23.4	0.716
Fullness	5.6	8.5	0.750
Nausea (%)	35.5	61.7	0.003
Migrainous symptoms (%)[1]			
Headache	42.1	34.0	0.349
Phonophobia	16.8	10.6	0.321
Photophobia	14.0	8.5	0.339
Visual aura	3.7	0.0	0.179
Family history (%)	12.1	4.3	0.220
SN (%)	23.4	36.2	0.100
HSN (%)	15.0	38.3	0.001
DHR direction (%)			0.767
Negative	40.2	34.0	
SC origin	43.0	46.8	
Non-SC origin	16.8	19.1	
DHR SPV(°/s)			0.744
0	40.2	34.0	
0-5	40.2	42.6	
≥ 5	19.6	23.4	
DHR duration (s)			
0	40.2	34.0	
0-60	25.2	23.4	
≥ 60	34.6	42.6	
DHL direction (%)			0.358
Negative	37.4	25.5	
SC origin	48.6	57.4	
Non-SC origin	14.0	17.0	
DHL SPV(°/s)			0.029
0	37.4	25.5	
0-5	34.6	57.4	
≥ 5	28.0	17.0	
DHL duration(°/s)			0.142
0	37.4	25.5	
0-60	24.3	19.1	

≥ 60	38.3	55.	
RTR direction (%)			0.248
Negative	48.6	36.2	
SC origin	40.2	44.7	
Non-SC origin	11.2	19.1	
RTR SPV(°/s)			0.188
0	48.6	36.2	
0-5	37.4	53.2	
≥ 5	14.0	10.6	
RTR duration(°/s)			0.046
0	48.6	36.2	
0-60	16.8	8.5	
≥ 60	34.6	55.3	
RTL direction (%)			0.150
Negative	48.6	34.0	
SC origin	41.1	46.8	
Non-SC origin	10.3	19.1	
RTL SPV(°/s)			0.171
0	48.6	34.0	
0-5	37.4	53.2	
≥ 5	14.0	12.8	
RTL duration(°/s)			0.162
0	48.6	34.0	
0-60	21.5	21.3	
≥ 60	29.9	44.7	
Horizontal gain			0.646
Normal	44.9	51.1	
Paranormal	42.1	40.4	
Subnormal	13.1	8.5	
Horizontal phase			0.321
Normal	39.3	29.8	
Paranormal	57.0	61.7	
Subnormal	3.7	8.5	
Asymmetry	18.7	19.1	0.947

[1]The following symptoms might co-exist.

[2]Hearing loss was symmetrical and mild to moderate in both ears with a past medical history.

References

1. Bhattacharyya, N.; Gubbels, S.P.; Schwartz, S.R.; Edlow, J.A.; El-Kashlan, H.; Fife, T.; Holmberg, J.M.; Mahoney, K.; Hollingsworth, D.B.; Roberts, R.; et al. Clinical Practice Guideline: Benign Paroxysmal Positional Vertigo (Update). *Otolaryngol Head Neck Surg* **2017**, *156*, doi:10.1177/0194599816689667.

2. Cohen, H.S. A review on screening tests for vestibular disorders. *J Neurophysiol* **2019**, *122*, 81-92, doi:10.1152/jn.00819.2018.
3. Falls, C. Videonystagmography and Posturography. *Adv Otorhinolaryngol* **2019**, *82*, 32-38, doi:10.1159/000490269.
4. Liu, D.; Wang, J.; Tian, E.; Guo, Z.-Q.; Chen, J.-Y.; Kong, W.-J.; Zhang, S.-L. Diagnostic Value of the Vestibular Autorotation Test in Menière's Disease, Vestibular Migraine and Menière's Disease with Migraine. *Brain Sci* **2022**, *12*, doi:10.3390/brainsci12111432.
